# The Relationship between Nocturnal Enuresis and Obstructive Sleep Apnea in Children

**DOI:** 10.3390/children11091148

**Published:** 2024-09-23

**Authors:** María Andreu-Codina, Danica Nikolic-Jovanovic, Eduard Esteller, Núria Clusellas, Montserrat Artés, Javier Moyano, Andreu Puigdollers

**Affiliations:** 1 Department of Orthodontics, Faculty of Dentistry, Universitat Internacional de Catalunya, 08195 Barcelona, Spain; mariaandreu@uic.es (M.A.-C.); danicanikolic@uic.es (D.N.-J.); nuriaclusellas@telefonica.net (N.C.); martes@uic.es (M.A.); jmoyano@uic.es (J.M.); 2 ENT Department, Hospital Universitari General de Catalunya, 08195 Barcelona, Spain; eesteller@gmail.com

**Keywords:** children obstructive sleep apnea, nocturnal enuresis, adenotonsillectomy, dentofacial features, Pediatric Sleep Questionnaire

## Abstract

Background: The aim of this study is to determine the prevalence of nocturnal enuresis (NE) in children with obstructive sleep apnea (OSA), the effect of adenotonsillectomy (AT) and the width of the arches, and to compare them with control children without respiratory problems. Methods: Children from 2 to 12 years old were divided into three groups: children with OSA and NE (n = 51), children with OSA without NE (n = 79), and the control group (n = 168). NE was defined as at least one bedwetting incident per month. Arch widths were measured at the baseline and one year after. OSA was diagnosed by means of polysomnography, and the apnea-hypopnea index (AHI) was obtained. Parents completed the Pediatric Sleep Questionnaire (PSQ) to classify their children into those with and without NE. Results: NE was present in 39.2% of children with OSA compared to 28% in the control group (*p* = 0.04). After AT, 49% of the children with OSA and NE significantly improved. Both OSA groups had narrower arch widths than the control group (*p* = 0.012), with the NE group having the narrowest widths. NE is more prevalent in children with OSA and should be considered one of the first signs of breathing disorders. Adenotonsillectomy reduces NE in about half of the affected children. Both arch widths are narrower in children with OSA, particularly in those with NE.

## 1. Introduction

Obstructive sleep apnea (OSA) is a sleep breathing disorder characterized by repeated episodes of upper airway obstruction during sleep, disrupting normal patterns (American Thoracic Society) [1,2]. The prevalence of childhood OSA diagnosed by varying criteria was reported to be 1% to 4% [3]. Some previous investigations stated that approximately 10% of the pediatric population regularly snore and that 1.2–5.7% suffer from OSA [3]. The peak incidence occurs between 3 and 6 years old, when the lymphatic tissue is the largest considering the underlying volume, and in pre-puberal children [4]. Impaired sleep due to OSA can lead to significant health issues in the pediatric population such as poor school performance, neurocognitive deficits, metabolic abnormalities, cardiovascular problems, and impaired craniofacial development [5,6,7,8,9,10,11,12,13].

Early studies have shown a relationship between nocturnal breathing problems and malocclusion, impacting craniofacial development [11]. Moss’s functional matrix theory suggests that nasal breathing is essential for proper dentofacial growth [14]. Continuous nasal airflow stimulates lateral maxillary growth and lowers the palatal vault [15]. Individuals with OSA, who have a narrowed upper airway and increased resistance [16,17], often exhibit dental abnormalities like narrow, v-shaped maxillary arches, high palatal vaults, and posterior crossbite [7,15,18,19,20].

Oral breathing alters neuromuscular activity, causing functional demands on craniofacial muscles, leading to malocclusion [19,21]. These patients typically have a short, retropositioned mandible, midface depression, backward tongue displacement, and a narrow, deep palate [6,11,15,17,22,23].

Polysomnography is the gold standard for detecting nocturnal breathing problems; in addition, the Pediatric Sleep Questionnaire (PSQ) is a valuable initial screening tool [24]. This questionnaire has a positive predictive value of 0.4 (i.e., 40% of patients with a positive PSQ score will be diagnosed with OSA) and a negative predictive value of 0.99 (i.e., only 1% of patients with a negative PSQ score will be diagnosed with OSA). The PSQ consists of 22 questions of yes/no/I do not know answers [25].

Moreover, adenotonsillectomy (AT) is the primary treatment for pediatric OSA, normalizing respiratory parameters and reversing complications in many cases [12,13]. Orthodontic treatments like skeletal palatal expansion and myofunctional therapy are also used [24].

Nocturnal enuresis (NE) is defined as intermittent incontinence during sleep, and its prevalence decreases with age [26,27,28]. To diagnose NE, a child aged 5 to 6 years must experience two or more bed-wetting episodes per month, while a child older than 6 years should have one or more episodes per month. NE is a common concern in 2–15% of children [26]; its prevalence in children globally varies between 2% and 10% depending on the study. However, in children with OSA, the prevalence of NE can be significantly higher, ranging from 10% to 40% [29,30,31]. The development of NE in children with OSA is complex and influenced by various factors. The exact biological mechanisms connecting NE and OSA remain unclear. Although issues like sleep arousal disturbances, bladder instability, and hormonal or metabolic imbalances may play a role, additional factors such as family environment, socioeconomic status, parental education levels, and physiological factors are significant contributors to NE in children with OSA. These highlight the need for a thorough, interdisciplinary evaluation of children with both NE and OSA to identify potential underlying causes and develop the most effective treatment strategies [32,33,34].

Nocturnal enuresis often resolves when OSA is treated through AT or palatal expansion. However, limited information exists on the prevalence of NE in OSA children compared to their healthy peers and its association with dentofacial issues or age-related differences. The aim of this study was to determine the prevalence of NE in children with apnea OSA, the effect of AT and the width of the arches, and to compare them with control children without respiratory problems, divided into two age groups: 2–<5 years and ≥5–12 years [35,36,37,38,39,40,41,42,43,44,45].

The main working hypothesis of this study was that NE would be more prevalent in the children with OSA and that they have narrower dental arches than the control children. On the other hand, the null hypothesis was that the presence of NE and the width of dental arches would be similar in OSA and control children.

## 2. Materials and Methods

This study received approval from the members of the Research Ethical Committee (CEIm) at the University Dental Clinic of Universitat Internacional de Catalunya, study code ORT-ECL-2010-01-NF, on 10 February 2022. Participants’ parents or legal guardians were informed about the procedures, and data collection was carried out both orally and in written form. Informed consent was obtained from all the participants.

The study sample consisted of 298 children in total within an age range of 2 to 12 years old. Participants were divided into two main groups: an experimental group (n = 130) and a control group (n = 168). The experimental group was further divided into two subgroups: children with OSA and NE (n = 51) and children with OSA but without NE (n = 79).

Children in the experimental group were recruited from the ENT department of the General Hospital of Catalunya, where they were diagnosed with OSA, as confirmed by polysomnography. All these patients had enlarged tonsils and/or adenoids and were candidates for AT. This group included 69 boys and 61 girls, with a mean age of 4 years and 9 months.

The control group was composed of children without respiratory problems, as confirmed by the negative results on the PSQ, who were recruited from the University Dental Clinic at Universitat Internacional de Catalunya and an accredited private clinic. This group involved 85 boys and 83 girls, with a mean age of 7 years and 4 months.

Patients who were excluded from the study included those whose parents or legal guardians did not consent to their participation; those who wore or had worn orthodontic or orthopedic appliances; those who had previously undergone AT or tonsillectomy; and children with craniofacial malformations, dental agenesis or supernumerary teeth.

This prospective quasi-experimental study involved a complete otorhinolaryngological examination and a polysomnographic diagnosis to determine the apnea-hypopnea index (AHI) in the OSA group. After the polysomnography, we could classify the apneas into mild (AHI ≥ 1–<5/h), moderate (AHI ≥ 5–<10/h), and severe (AHI ≥ 10/h). Question number 9 of the PSQ was used to check if the child presented with NE and was completed by the parents with the advice of an expert in sleep medicine.

Following the initial assessment (T0), children in the OSA group underwent adenotonsillectomy performed by an otolaryngologist. One year after the surgery, these children were re-evaluated by the ENT department with a polysomnography and the same questionnaire (T1). Parents of the control group children completed the same questionnaire at the same time intervals, both at baseline and the 1 year follow-up.

Dentofacial parameters, both intercanine and intermolar widths of the upper and the lower arch, were measured in all participants at T0 and T1. The measures were performed on stone casts using calipers measuring the distances between the tips of the canines for the intercanine width and the distance between the mesiobuccal cusp tips of the first molars for the intermolar distance. All the measurements were performed by were examiners and repeated twice.

Data was recorded in an Excel (Microsoft Corporation, Microsoft Excel 365 version 16.89, Redmond, WA, USA) spreadsheet, and statistical analysis was performed using R software version 4.1.2. Descriptive statistics were calculated for all study variables, including means, standard deviations, medians and percentiles of numeric data, and counts and percentages for categorical variables. To test the normality of the sample, the Shapiro–Wilk test was conducted. Levene’s test was used to assess the homogeneity of variances. For independent variables that followed a normal distribution, Student’s *t*-test was performed. For data that did not follow a normal distribution, the Mann–Whitney U non-parametric test was applied. Categorical responses were analyzed using the Chi-Squared test (X^2^). The Chi-Squared test was used to compare NE prevalence between the OSA and control groups, while the Mann–Whitney U non-parametric test was used to assess changes in NE prevalence within groups over time (pre- and post-surgery for the OSA group and T0 vs. T1 for the control group). The relationship between age and nocturnal enuresis was assessed using the Chi-Squared test. It was also used to compare the severity of apnea (mild, moderate, severe) between children with and without NE both before and after AT. The comparison of dentofacial features was conducted using either the Student’s *t*-test or the Mann–Whitney U non-parametric test, depending on the data distribution.

The differences between patients with and without NE were statistically evaluated. Effect size estimates included the relevant 95% confidence intervals. Statistical significance was accepted at a *p*-value < 0.05 for all analyses.

## 3. Results

### 3.1. Sample Demographics

This study included 130 subjects in the OSA group, while the control group consisted of 168 subjects. Both groups were matched samples in sex and age.

Sex was not a significant factor in the occurrence of OSA, as the results showed no statistical difference between boys and girls. However, age was significantly related to OSA, with a higher percentage of OSA observed in children under 5 years old (*p* < 0.001) (Table 1).

### 3.2. Prevalence of Nocturnal Enuresis

Regarding NE, in the OSA group, 39.2% of patients experienced NE compared to 28% in the control group. In the current sample, the OSA group showed a significantly higher prevalence of NE than the control group (*p* = 0.04).

As seen in Table 2, before adenotonsillectomy, 51 children (39.2%) with OSA had NE. After surgery, this number deceased to 26 (20%) (*p* < 0.001), indicating a significant reduction in NE prevalence. Specifically, 49% of the patients with both OSA and NE experienced a complete resolution of NE after surgery.

In the control group, 47 children (28%) had NE at T0, while one year after at T1, 28 children (16.7%) had NE (*p* < 0.001), which is a significant decrease (Table 2).

At T0, the prevalence of NE between the OSA group and the control group was significantly different (*p* = 0.040). However, at T1, there were no significant differences in the prevalence of NE (Table 3).

At both T0 and T1, children with OSA had a higher prevalence of NE compared to the control group, with the results being statistically significant.

These results indicate a significant reduction in NE prevalence after adenotonsillectomy, with a notable improvement in NE symptoms in both groups, though more pronounced in the OSA group.

### 3.3. Influence of Age on Nocturnal Enuresis

Age was found to be related to the presence of NE. In the OSA group at T0, 41.6% of the children under 5 years had NE compared to 35.8% of the children aged 5 years and older. At T1, 22.2% of the children under 5 years had NE, while 18.4% of the children aged 5 years and older had NE. However, these results were not statistically significant (Table 4).

In the control group, slight differences were observed between the two age groups. At T0, 32.5% of the children under 5 years had NE compared to 26.6% of the children aged 5 years and older. At T1, 24.2% of the children under 5 years had NE, while 14.8% aged 5 years and older had NE. Children under 5 years in the control group had a higher prevalence of NE (Table 5).

Thus, it was observed that in both the control and OSA groups, the age group under 5 years had a higher percentage of NE at both T0 and T1. However, these results were not statistically significant.

### 3.4. Apnea-Hypopnea Index (AHI) Measurements

Patients with OSA, with and without NE, showed very similar AHI results, though those with NE had slightly higher AHI values.

At T0, among the children with NE, 51% had mild, 25.5% had moderate, and 23.5% had severe apnea. For children without NE, 50.6% had mild, 38% had moderate, and 11.4% had severe apnea.

In both groups with and without NE, the severity and prevalence of apnea decreased significantly after adenotonsillectomy. For children with NE, at T1, 30.8% had no apnea and 69.2% had mild apnea; none presented with moderate or severe apnea. Among the children without NE, at T1, 20.2% had no, 72.8% had mild, 5.0% had moderate, and 2.0% had severe apnea. Note that at T1, there were five patients who had no AHI recorded as they were pending polysomnography.

The results of the apnea-hypopnea index (AHI) variable are presented in Table 6.

### 3.5. Dentofacial Features

When comparing dentofacial features, children with OSA exhibited narrower intercanine and intermolar widths in both the upper and lower arches at T0 and T1 compared to the control group.

The measurements of the widths, both upper and lower arch, can be seen in Table 7.

At both T0 and T1, the arch widths of the maxilla and mandible in the OSA group were narrower before and after AT in comparison with the arch widths of the control group.

These differences highlight the impact of OSA on dentofacial development, with children with OSA showing consistently narrower dental arch widths than healthy children, even after surgical intervention. This suggests that while adenotonsillectomy may alleviate some symptoms of OSA, its effects on dentofacial structure may require additional orthodontic or orthopedic treatment.

Remarkably, within the OSA group, children with OSA and NE had even narrower intermolar widths than those without NE at T0, with these differences being statistically significant (Table 8).

## 4. Discussion

The aim of this study was to investigate the relationship between OSA and NE in children.

In this study, a significant association was observed between OSA and NE. The prevalence of NE was 39.2% in children with OSA compared to 28% in the control group. This indicates that children with OSA have a higher likelihood of experiencing NE. Statistical analysis confirmed this association as significant (*p* = 0.04), highlighting the importance of considering OSA when evaluating and managing children with NE.

These results align with the findings from previous studies. Tsai et al. [44] reported an increased prevalence of NE in children with OSA (*p* < 0.001). Park et al. [42] also found a higher prevalence of NE in children with OSA than in healthy children (*p* = 0.036). Lumeng showed that children with severe OSA had a significantly increased frequency of bed-wetting (*p* = 0.003) and that children with NE were significantly more likely to have OSA [45]; Alexopoulos found that 16.6% of 355 children with NE had moderate-to-severe OSA [46]. Brooks and Topol [39] noted that 41% of 115 children with OSA exhibited NE, closely mirroring our data, with an enuretic rate of 39.2% in the OSA group. Moreover, Basha [38] confirmed that the association between NE and OSA is real and has a reasonable physiological basis, recommending that OSA should be considered in the differential diagnosis of NE in children.

However, some studies have found no relationship between OSA and NE. In the study by Ng et al. [47], it was reported only a prevalence of 5.1% of NE out of a large sample of 3047 children in total. The difference in the results obtained compared to those of our study may be due to their sample consisting of children aged 6 to 12 years old and their definition of NE as more than one episode per week. Additionally, and in contrast to our study, Miao Shang Su [48] did not find a higher NE prevalence in children with OSA but noted a differential sex response in NE prevalence with increasing OSA severity. They studied 6147 children aged 6 to 11 years, defining NE as more than one wet night per month. The overall prevalence of NE was 4.6% (6.7% of boys and 2.5% girls), with no significant difference between children with and without OSA. The differences in findings between their study and ours may be attributed to their older patient sample and different NE definition.

The relationship between NE and OSA is controversial, with studies reporting both positive and negative associations. These discrepant findings might be related to different study designs and varying sample sizes.

Our study found no statistically significant differences in the prevalence of OSA and NE between boys and girls, indicating that gender does not influence the occurrence of these conditions. This contrasts with Su’s findings, which noted a higher prevalence in boys. Additionally, our results show that children under 5 years old, in both the OSA and control groups, had a higher prevalence of NE compared to other aged children. This aligns with the natural decrease in NE as children age, due to the maturation of bladder control and neurological development. Su also observed a decline in NE prevalence with age, from 9.0% at age 6 to 1.9% at age 11. Younger children tend to have more NE due to delayed maturation of the nervous system, bladder function, and reduced nighttime secretion of the antidiuretic hormone [48].

In this research, we found that after adenotonsillectomy, 49% of children with OSA and NE experienced an improvement in NE, compared to 40.4% in the control group at T1. This suggests that surgical intervention had a more positive impact on NE in the OSA group. Approximately half of the children with OSA and NE had a resolution of NE after surgery. Similar results were reported by other studies. Park found that NE decreased from 9.3% before surgery to 1.5% after surgery in children with OSA [42]. Thottam observed that more than half (51.4%) of children experience NE resolution post-surgery, especially those with a higher apnea-hypopnea index [49]. Jeyakumar’s retrospective study also showed NE improvement in children with OSA who underwent adenoidectomy and/or tonsillectomy [37].

Su’s systematic review in 2019 confirmed that surgery correcting upper respiratory tract obstruction generally improves OSA and NE symptoms in affected children [31]. Weider et al. found a 76% improvement in NE symptoms post-surgery among 115 children with NE and OSA [50]. Basha reported an 84,2% improvement rate, with 61.4% experiencing complete elimination of NE and 22.8% showing significant symptom reduction [38]. However, Davaro’s recent study of 59 Caucasian patients, with an average age of 8.8 years, found that surgical treatment had no effect on NE resolution in patients with OSA and NE [51]. Though these results are interesting, the study was limited by its small sample size.

In our study, patients with NE had a slightly higher AHI compared to those without NE, but this difference was not statistically significant. Similar to our results, in Alexopoulos’s large cohort study of hospital-referred children with habitual snoring, a significant association between NE and moderate-to-severe OSA was found, regardless of sex [46].

Despite numerous articles exploring the relationship between OSA and NE, many studies have been constrained by small sample sizes, recruitment solely from sleep centers, inadequate control for participant age and disease definition, and insufficient matching of participants in control groups.

The observation of NE in children should be considered a clear sign of respiratory problems, and it is important that pediatricians and orthodontists rule out the presence of nocturnal apnea.

The clinical implications of the present study underscore the importance of recognizing NE as a common finding in children with OSA symptoms. Consequently, OSA should be considered in the differential diagnosis of a child presenting with NE, and vice versa. Both for doctors and orthodontists, these findings suggest the following practical recommendation: include OSA in the differential diagnosis for children presenting with NE. The early identification of OSA can lead to timely and effective treatment, potentially improving NE symptoms.

The use of the PSQ routinely by pediatricians, GPs, and orthodontists during initial evaluations to identify potential OSA cases is highly recommended. If there is a suspicion of OSA, regarding question number 9 on the PSQ or a positive response concerning the signs and symptoms of OSA, promptly refer the child to an otorhinolaryngologist for further assessment.

Parents should be informed that NE has a 50% chance of improvement and resolution post-tonsillectomy, and a clear understanding of the potential benefits of the surgery should be provided. And, a further and very important issue is psychological support when the child stops wetting at night. Additionally, orthodontic treatments, such as maxillary expansion, should be considered as potential supplementary approaches to improve upper airway flow, particularly beneficial in children with narrow maxillary arches. However, it is important to keep in mind that orthodontic treatment can be prescribed only if there is a real orthodontic problem such as maxillary compression. As seen in a latest review on novel therapies for preventing, managing, and treating OSA and snoring in pediatric and adult patients [52], it was concluded that there was insufficient evidence to support RME as a treatment to cure pediatric OSA, and expansion should only be considered in patients who demonstrate maxillary constriction, independent of having OSA.

Nevertheless, this study deserves some considerations and the pondering of some limitations. First, the sample was non-random, consisting of consecutive patients treated according to their disease severity, which could not be randomized due to ethical considerations. Second, the PSQ was used to rule out apnea in the control group, because the negative predictive value of the test is 99%. Third, regarding the sample, there are two limitations: the difference in sample size and the non-response rate of 15% to our PSQ. This non-response may indicate selection bias, as parents of children with sleep symptoms were more likely to return the questionnaire. Additionally, dropouts led to a smaller sample at T1 than at T0, primarily because some parents refused a follow-up polysomnography once their children were healthy.

Moreover, the completion of the questionnaire by parents who often did not share a sleeping space with their child posed another limitation. This lack of direct observation could have affected the accuracy of their responses regarding their child’s sleep behavior. Also, it is common that parents tend to hide the NE of their children if they filled out the questionnaires when their offsprings were around. This limitation is commonly associated with the use of survey questionnaires.

On the contrary, regarding the strengths of this study, although NE is considered in children above five years old, we included a sample of OSA children and controls of two to five years old because we wanted to check if NE was more prevalent in children with OSA than in controls not only at the common age for OSA, but also at an early age starting from two years old. These data reinforce the importance of an early diagnosis of checking if AT helps improve NE in this younger group of children, and of checking if any craniofacial development constriction can be observed.

Furthermore, OSA was diagnosed with polysomnography, the gold standard, and further classified as mild, moderate, or severe, with a follow-up one year after adenotonsillectomy.

Despite these limitations and considerations, our study provides valuable insights into the relationship between OSA and NE in children. Future research should aim to include larger samples and consider alternative methods to gather more accurate sleep behavior data.

## 5. Conclusions

Based on our findings, we can draw the following conclusions:

- Nocturnal enuresis can be considered one of the first signs of OSA in children.
- The prevalence of NE is significantly higher in children diagnosed with OSA compared with healthy children.
- Adenotonsillectomy led to improvements in NE symptoms in half of the children who underwent the surgery.
- Children with NE had a higher AHI, although this association did not reach statistical significance.
- Children diagnosed with OSA exhibited narrower dental arches than healthy children, with those with OSA and NE showing even narrower intermolar widths. It is important to perform interdisciplinary assessments to develop the most appropriate treatment options for the best therapeutic effect.

These conclusions underscore the importance of recognizing NE as a potential clinical marker of OSA in pediatric patients, highlighting the need for early intervention and appropriate management strategies to improve patient outcomes.

## Figures and Tables

**Table 1:** Sample demographics of OSA and control children by sex and in both age groups: 2–<5 years and ≥5–12 years. (* *p*-value < 0.05).

		OSA N = 130		Control N = 168		Total N = 298		*p*-Value
		Frequency	Percentage	Frequency	Percentage	Frequency	Percentage	
			(%)		(%)		(%)	
Sex	Male	69	53.1	85	50.6	154	100	
	Female	61	46.9	83	49.4	144	100	
	Total	130	100	168	100	298	100	0.671
Age group	2–<5	77	59.2	40	23.8	117	100	
	≥5–12	53	40.8	128	76.2	181	100	
	Total	130	100	168	100	298	100	<0.001 *

**Table 2:** Intra-group comparison of the prevalence of NE at T0 and T1 in both OSA and control groups. (* *p*-value < 0.05).

		NE		No NE		*p*-Value
		Frequency	Percentage	Frequency	Percentage	
			(%)		(%)	
OSA	T0	51	39.2	79	60.8	
	T1	26	20.0	104	80.0	<0.001 *
Control	T0	47	28.0	121	72.0	
	T1	28	16.7	140	83.3	<0.001 *

**Table 3:** Inter-group comparison of the prevalence of NE at T0 and T1 in both OSA and control groups. (* *p*-value < 0.05).

		NE		No NE		*p*-Value
		Frequency	Percentage	Frequency	Percentage	
			(%)		(%)	
T0	OSA	51	39.2	79	60.8	
	Control	47	28.0	121	80.0	0.040 *
T1	OSA	26	20.0	104	80.0	
	Control	28	16.7	140	83.3	0.459

**Table 4:** Comparison of presence of NE in OSA children in both age groups: 2–<5 years and ≥5–12 years in T0 and T1.

OSA	Group							
	Age Group	Total		NE		No NE		*p*-Value
		Frequency	Percentage (%)	Frequency	Percentage (%)	Frequency	Percentage (%)	
T0	2–<5	77	59.2	32	41.6	45	58.4	
	≥5–12	53	40.8	19	35.8	34	64.2	0.512
T1	2–<5	54	41.5	12	22.2	42	77.8	
	≥5–12	76	58.5	14	18.4	62	81.6	0.593

**Table 5:** Comparison of presence of NE in control children in both age groups: 2–<5 years and ≥5–12 years in T0 and T1.

Control	Group							
	Age Group	Total		NE		No NE		*p*-Value
		Frequency	Percentage (%)	Frequency	Percentage (%)	Frequency	Percentage (%)	
T0	2–<5	40	23.8	13	32.5	27	57.5	
	≥5–12	128	76.2	34	26.6	94	73.4	0.465
T1	2–<5	33	19.6	8	24.2	25	75.8	
	≥5–12	135	80.4	20	14.8	115	85.2	0.193

**Table 6:** Comparison of AHI findings between children with NE and without NE in the OSA group.

	AHI	with NE	Group	without NE	Group	Total		*p*-Value
		Frequency	Percentage (%)	Frequency	Percentage (%)	Frequency	Percentage (%)	
T0	None	0	0.0	0	0.0	0	0.0	
	Mild Moderate Severe Total	26 13 12 51	51.0 25.5 23.5 100	40 30 9 79	50.6 38.0 11.4 100	66 43 21 130	50.8 33.1 16.1 100	0.117
T1	None	8	30.8	20	20.2	28	21.5	
	Mild Moderate Severe Not registered Total	18 0 0 - 26	69.2 0 0 - 100	72 5 2 - 99	72.8 5.0 2.0 - 100	90 5 2 5 130	69.2 3.9 1.5 3.9 100	0.173

**Table 7:** Comparison of dentofacial measures between OSA group and control group at T0 and T1. (* *p*-value < 0.05).

**T0 Dentofacial measures**							
		OSA			Control		*p*-value
	Frequency	Mean	St Dev	Frequency	Mean	St Dev	
Upper intercanine width	122	27.52	2.81	105	30.51	2.77	0.000 *
Lower intercanine width	122	22.92	2.17	107	24.79	2.25	0.000 *
Upper intermolar width	121	33.19	3.45	111	37.30	4.10	0.000 *
Lower intermolar width	122	33.39	3.52	110	37.71	3.86	0.000 *
**T1 Dentofacial measures**							
		OSA			Control		*p*-value
	Frequency	Mean	St Dev	Frequency	Mean	St Dev	
Upper intercanine width	61	28.78	2.81	102	30.97	2.67	0.000 *
Lower intercanine width	61	23.61	2.27	103	25.12	2.33	0.000 *
Upper intermolar width	59	34.24	3.89	111	37.95	4.06	0.000 *
Lower intermolar width	59	35.24	3.30	110	37.93	3.82	0.000 *

**Table 8:** Comparison of dentofacial measures in OSA group with and without NE at T0. (* *p*-value < 0.05).

**OSA group** **T0 Dentofacial measures**							
		NE			No NE		*p*-value
	Frequency	Mean	St Dev	Frequency	Mean	St Dev	
Upper intercanine width	47	27.28	2.96	75	27.67	2.73	0.461
Lower intercanine width	48	22.85	2.52	74	22.96	1.92	0.791
Upper intermolar width	47	32.19	3.81	74	33.83	3.05	0.015 *
Lower intermolar width	49	33.42	3.30	78	37.93	3.82	0.011 *
**OSA group** **T1 Dentofacial measures**							
		OSA			Control		*p*-value
	Frequency	Mean	St Dev	Frequency	Mean	St Dev	
Upper intercanine width	13	28.56	2.93	48	28.85	2.80	0.757
Lower intercanine width	13	23.59	2.78	48	23.62	2.14	0.978
Upper intermolar width	12	31.84	4.86	47	34.85	3.40	0.064
Lower intermolar width	12	33.49	3.49	47	35.69	3.10	0.071

## Data Availability

The data presented in this study are available upon request from the corresponding author. The data are not publicly available due to patient privacy.
